# The p75^NTR^ neurotrophin receptor is required to organize the mature neuromuscular synapse by regulating synaptic vesicle availability

**DOI:** 10.1186/s40478-019-0802-7

**Published:** 2019-09-12

**Authors:** Viviana Pérez, Francisca Bermedo-Garcia, Diego Zelada, Felipe A. Court, Miguel Ángel Pérez, Marco Fuenzalida, Johanna Ábrigo, Claudio Cabello-Verrugio, Guillermo Moya-Alvarado, Juan Carlos Tapia, Vicente Valenzuela, Claudio Hetz, Francisca C. Bronfman, Juan Pablo Henríquez

**Affiliations:** 1 Neuromuscular Studies Laboratory (NeSt Lab), Department of Cell Biology, Center for Advanced Microscopy (CMA BioBio), Universidad de Concepción, Concepción, Chile; 2 Center for Integrative Biology, Faculty of Sciences, Universidad Mayor; FONDAP Center for Geroscience, Brain Health and Metabolism, Santiago, Chile; 3 Laboratory of Neural Plasticity, Center for Neurobiology and Integrative Physiology, Faculty of Sciences, Institute of Physiology, Universidad de Valparaíso, Valparaíso, Chile; 4 Present Address: Health Sciences School, Universidad de Viña del Mar, Viña del Mar, Chile; 5 Laboratory of Muscle Pathologies, Fragility and Aging, Department of Biological Sciences, Faculty of Life Sciences, Millennium Institute on Immunology and Immunotherapy, Universidad Andrés Bello, Santiago, Chile; 6 Department of Physiology, Faculty of Biological Sciences, Pontificia Universidad Católica de Chile, Santiago, Chile; 7 Department of Biomedical Sciences, Faculty of Health Sciences, Universidad de Talca, Talca, Chile; 8 Biomedical Neuroscience Institute, Faculty of Medicine, University of Chile, Santiago, Chile; 9 Center for Geroscience, Brain Health and Metabolism, Santiago, Chile; 10 Program of Cellular and Molecular Biology, Institute of Biomedical Sciences, University of Chile, Santiago, Chile; 11 Buck Institute for Research on Aging, Novato, CA 94945 USA; 12 Center for Aging and Regeneration (CARE), Institute of Biomedical Sciences (ICB), Faculty of Medicine and Faculty of Life Sciences, Universidad Andrés Bello, Santiago, Chile

**Keywords:** Neuromuscular junction, p75^NTR^, Neurotrophin, Motor neuron, Synaptic vesicles

## Abstract

The coordinated movement of organisms relies on efficient nerve-muscle communication at the neuromuscular junction. After peripheral nerve injury or neurodegeneration, motor neurons and Schwann cells increase the expression of the p75^NTR^ pan-neurotrophin receptor. Even though p75^NTR^ targeting has emerged as a promising therapeutic strategy to delay peripheral neuronal damage progression, the effects of long-term p75^NTR^ inhibition at the mature neuromuscular junction have not been elucidated. We performed quantitative neuroanathomical analyses of the neuromuscular junction in p75^NTR^ null mice by laser confocal and electron microscopy, which were complemented with electromyography, locomotor tests, and pharmacological intervention studies. Mature neuromuscular synapses of p75^NTR^ null mice show impaired postsynaptic organization and ultrastructural complexity, which correlate with altered synaptic function at the levels of nerve activity-induced muscle responses, muscle fiber structure, force production, and locomotor performance. Our results on primary myotubes and denervated muscles indicate that muscle-derived p75^NTR^ does not play a major role on postsynaptic organization. In turn, motor axon terminals of p75^NTR^ null mice display a strong reduction in the number of synaptic vesicles and active zones. According to the observed pre and postsynaptic defects, pharmacological acetylcholinesterase inhibition rescued nerve-dependent muscle response and force production in p75^NTR^ null mice. Our findings revealing that p75^NTR^ is required to organize mature neuromuscular junctions contribute to a comprehensive view of the possible effects caused by therapeutic attempts to target p75^NTR^.

**Electronic supplementary material:**

The online version of this article (10.1186/s40478-019-0802-7) contains supplementary material, which is available to authorized users.

## Introduction

The vertebrate neuromuscular junction (NMJ) is a peripheral cholinergic synapse formed by a motor axon terminal and a skeletal muscle fiber specialization, a structure capped by terminal Schwann cells. During embryonic NMJ development, pre and postsynaptic signals regulate both the clustering of acetylcholine receptors (AChRs) on the muscle membrane and the subsequent innervation of nascent postsynaptic domains. During NMJ maturation, early post-natal elliptical postsynaptic *plaques* gradually re-organize to form *pretzel*-like structures, complex arrangements containing regions of high and low AChR density [5, 46, 54]. At the ultrastructural level, AChRs concentrate at the edges of secondary folds, which are localized in direct apposition to the presynaptic active zones containing synaptic vesicle clusters and membrane proteins that allow for efficient neurotransmitter release [71, 90]. Even though presynaptic stimulation and transmitter release are required for postsynaptic maturation at the NMJ [54], the molecular signals controlling the architecture of functional mature NMJs have not been fully elucidated.

Neurotrophins (NTs) are a family of growth factors that play a wide variety of functions in the nervous system through their binding to and activation of specific tyrosine kinase receptors (Trks) [10]. Effects on neuronal survival and neuronal growth are mainly triggered by binding of the nerve growth factor (NGF) to TrkA, the brain-derived neurotrophic factor (BDNF) and NT-4 to TrkB, and the NT-3 to TrkC [11, 33]. In addition, all NTs and their non-processed forms (pro-NTs) bind to the pan-NT receptor p75 (p75^NTR^), a multifunctional signaling receptor that belongs to the tumor necrosis factor receptor family [10]. Inhibition of axonal pruning, long-term depression and developmental or injury-induced apoptosis mainly rely on the binding of NTs and pro-NTs to p75^NTR^ [22, 85, 93].

While p75^NTR^ is widely expressed in different neuronal and glial populations in the developing nervous system, its expression is down-regulated towards adulthood [57, 95]. All three NMJ cellular components retain low p75^NTR^ expression levels at adult stages [25–27]. Even though p75^NTR^ null mice (p75^NTR^−/−) display delayed NMJ synaptic refinement during early post-natal development, these phenotypes become soon restored [37]. Remarkably, p75^NTR^ is strongly re-expressed in motor neurons and Schwann cells in conditions that negatively affect the nervous system [35]. These include experimental paradigms of nerve injury [39, 65, 79, 89, 91] and of amyotrophic lateral sclerosis [41, 52, 72], a neurodegenerative disease characterized by NMJ disruption and subsequent motor neuron death [59]. Cumulative evidence has demonstrated that p75^NTR^ up-regulation impairs nervous system repair [2, 20, 35, 79, 88] and, consistently, p75^NTR^ targeting has emerged as a therapeutic alternative to delay damage or disease progression [56, 74, 81]. Even though the aforementioned evidence reveals that p75^NTR^ targeting could be beneficial for nerve repair, the effects of chronic p75^NTR^ inhibition at the mature neuromuscular synapse have not been deeply analyzed.

Our goal was to perform in-depth neuroanathomical and neurophysiological analyses of the NMJ of p75^NTR^−/− mice [48]. In these mice, we found altered NMJ morphology, evidenced by decreased size and aberrant postsynaptic organization. These phenotypes were accompanied by increased muscle fatigability after presynaptic stimulation, reduced muscle fiber size and locomotor defects. Also, p75^NTR^−/− mice display a reduced number of synaptic vesicles in motor axon terminals. Functional experiments showed that acetylcholinesterase inhibition rescued nerve-evoked muscle response and force production. Together, these studies reveal that the absence of p75^NTR^ negatively affects NMJ neurotransmission, which correlates with impaired morphology and function of the neuromuscular synapse.

## Materials and methods

### Animals

The C57BL/6 J strain B6.129S4-Ngfrtm1Jae/J p75^NTR^−/− mice [48] and their control pairs were purchased from The Jackson Laboratory (Sacramento, California, USA). All in vivo tests were carried out in procedure rooms equipped for that purpose. Mice were fed with pellet (Prolab RMH-3000, LabDiet) and water ad libitum and sacrificed by inhalatory isofluorane anesthesia overdose and posterior cervical dislocation, when indicated. Our procedures have been approved by the Bioethics Committee at the University of Concepcion, Chile, and follow the rules imposed by the Bioethics Committee of the National Commission for Scientific and Technological Research, Chile (CONICYT), and have therefore been performed in accordance with the ethical standards laid down in the Animals (Scientific Procedures) Act 1986, UK.

### NMJ staining and analyses

Diaphragm and *Levator auris longus* (LAL) muscles were dissected and whole-mount fixed in 0.5% formaldehyde (FA) in 1X Phosphate buffered saline (PBS) at 22 °C for 90 min. Samples were incubated with 0.1 M glycine in 1X PBS, permeabilized with PBST (1X PBS/0.5% TritonX-100) and blocked with 4% Bovine serum albumin (BSA) dissolved in PBST 12-16 h at 4 °C. Muscles were incubated with mouse monoclonal antibodies raised against neurofilament (2H3) (1:300) and synaptic vesicles (SV2) (1:50) (both from the Developmental Studies Hybridoma Bank, DSHB, of the University of Iowa, USA) along with a rabbit anti S100 antibody (1:300) (DAKO, Santa Clara, CA, USA) in 4% BSA-PBST for 30 min at RT and then 12-16 h at 4 °C. The tissues were incubated with the respective secondary antibodies (1:300) (Jackson Immuno Research, West Grove, PA, USA) in 4% BSA-PBST containing Alexa488-conjugated α-bungarotoxin (BTX) (Invitrogen, Carlsbad, CA, USA) (1:500) and DAPI (1:1000) (Thermo Fisher, Waltham, MA, USA) 12-16 h at 4 °C. Samples were post-fixed with 1% FA in 1X PBS for 10 min at 22 °C, flat mounted between two coverslips, and imaged. To analyze NMJ morphological maturation, *z*-stack images were collected at 1 μm intervals in a Zeiss LSM 780 confocal microscope at the CMA Bio-Bio facility, University of Concepcion, Chile. Maximal intensity projection images were reconstructed in 3D using the ImageJ software and analyzed to determine the proportion of the different postsynaptic NMJ morphologies, which were grouped into those having a single peripheral opening (still maturing) or those having multiple peripheral openings (mature pretzels). The morphology of > 130 NMJs per mice was manually determined and expressed as the percentage of the total. Images were obtained by processing confocal z-stack images using Imaris software. The surface, volume, and area of > 50 AChR densities per mice were determined for each postsynaptic structure using the ImageJ software, as described [40]. The LAL muscle innervation profile was analyzed in low magnification (10X) epifluorescence images (acquired with a Nikon Eclipse 80i microscope) of the entire rostral and caudal portions of the muscle, which were assembled using the ImageJ software. To determine NMJ innervation, confocal images were analyzed as described [40]. Briefly, the free-AChR presynaptic and the total AChR positive areas of > 35 NMJs per mice were calculated. Data are expressed as the fractional apposition between the pre- and postsynaptic domains. To analyze AChR dynamics, isofluorane anesthetized mice from both genotypes were subjected to a subcutaneous injection (in the head/neck region) of a non-saturating concentration (diluted 4 μg/mL in sterile 1X PBS) of Alexa-488 conjugated BTX (BTX-1). Subsequently, the right hemi-LAL muscle was denervated by facial nerve transection, as described [63]. Seven days post-surgery, mice were sacrificed and the LAL muscles were dissected, pinned to a sylgard dish and fixed with 0.5% v/v formaldehyde (Merck-Millipore) for 90 min at room temperature. After fixation, the muscles were washed and labeled with Alexa-555 conjugated BTX for 60 min (BTX-2, diluted 2 μg/mL in 1X PBS). Images (*z*-stacks) were collected at 1 μm intervals in a Zeiss LSM 700 confocal microscope at the CMA Bio-Bio facility (University of Concepcion, Chile). Following a blind assessment-based quantification, NMJs were categorized in “stable” if BTX-1 and BTX-2 labels were similarly intense, or as “dynamic” if BTX-1 labelling was mostly absent and BTX-2 intensity was comparatively higher. Data are expressed as the percentage of stable and dynamic denervated NMJ postsynaptic apparatuses in both genotypes [29].

### Transmission Electron microscopy

NMJ-enriched samples from the diaphragm muscle were fixed in 2.5% glutaraldehyde in 0.1 M phosphate buffer, pH 7.4, incubated with 1% Osmium tetroxide for 2 h and dehydrated with graded ethanol series. After embedding in EPON, 70 nm ultrafine sections were obtained, contrasted with uracyl acetate and lead citrate, and visualized in a transmission electronic microscope PHILIPS TECNAI 12 BIOTWIN [13]. Images were acquired with the following magnifications: 6000X, 16,500X, 26,500X, and 43,000X. Quantifications were performed as described [66]. Briefly, the number of active zones, the number of vesicles per terminal area, the diameter of vesicles, and the number of active zones without docked vesicles (defined as those having their membrane apposing the presynaptic membrane), were quantified as presynaptic parameters. The readily releasable pool (RRP) of vesicles, defined as the vesicles contained within a 480 nm wide strip of the terminal directly across from the synaptic cleft, was also determined. Quantified postsynaptic parameters included the postsynaptic perimeter, the number, depth and width of the secondary folds, as well as the width of the primary fold, defined as the synaptic cleft space. Postsynaptic parameters were divided by the total apposition length of the motor terminal to control for differences in the size of each analyzed junction. All measurements were performed using the ImageJ software, as described [66].

### Primary muscle culture

Myofibers from the gastrocnemius muscle were dissociated and incubated for 3–4 days in matrigel-coated (ThermoFisher Scientific) dishes. For enrichment of the myoblast population, adhered cells were trypsinized and pre-plated twice onto uncoated dishes for 1 h at 37 °C and 5% CO_2_. Cultures were maintained at less than 50% confluence in Bioamf-2 medium with 1% Pen/Strep at 37 °C and 5% CO_2_ [36]. Primary myoblasts were seeded onto cell culture plates coated with poly-ornithine and laminin in L^− 15^ medium, as described [45]. Cells were triggered to fuse into myotubes by incubation for 5 days with a differentiation medium containing 1X DMEM supplemented with 1% Glutamax-100, 1% Pen/Strep, 10% Fetal Bovine Serum, and 10% Horse Serum. AChR aggregates were labelled with Alexa488-conjugated BTX (1:500) for 45 min in culture conditions and the cells were subsequently fixed with 2% PFA for 20 min at 4 °C followed by incubation with 100% methanol for 5 min at − 20 °C. Cells were permeabilized with 0.1% TrisPO_4_/Triton X-100 and incubated with a mouse anti α-tubulin (1:1000) antibody (Sigma-Aldrich, St. Louis, MO, USA) in 1% TrisPO_4_ supplemented with 1% BSA 12-16 h at 4 °C. Cells were then incubated with the respective secondary antibodies (1:300) (Jackson Immuno Research) in 4% BSA-PBST containing Alexa488-conjugated BTX (1:500) and phalloidin (1:200) (Invitrogen) 2 h at 22 °C. Postsynaptic morphologies were categorized into “plaques”, small immature uniformly stained structures, and “complex” shapes, those comprising regions of low AChR density resulting in “O”, “C” and “pretzel-like” structures [45]. The myotube perimeter per field was manually traced on bright field images and their area was calculated using the ImageJ software. The proportions of the different postsynaptic structures in myotubes from both genotypes are expressed as a fraction of the total area of myotubes.

### Neuromuscular and muscle functional analyses

To analyze neuromuscular function, the effects of sciatic nerve stimulation frequency on muscle force-intensity and fatigue were studied after repetitive supramaximal stimulation protocols. Briefly, mice were anesthetized with ketamine (60 mg/kg) and xylazine (6 mg/kg) and an incision was made parallel to the femur bone to expose the sciatic nerve. A longitudinal skin incision was made to expose the Achilles tendon, which was separated from the tibia bone via blunt dissection, and firmly tied to a force transducer (UFI model 1030). Indirect stimulation was accomplished by placing bipolar electrodes in contact with the exposed sciatic nerve using a stimulator (model 611, Phipps and Bird, INC). Nerves were stimulated using 0.1 ms pulses, at supramaximal current intensity (5 mA) delivered at different frequencies. To induce fatigue, repetitive stimulations for 60s at 1, 10, and 100 Hz were performed. The different time phases of single twitch contractions as well as the contractile force and fatigue (force measurements as a percentage of maximal force) were analyzed using a Power Lab 4/35 device and the LabChart 8 software (AD Instruments, Dunedin, New Zealand). To measure skeletal muscle contractile properties, the Tibialis Anterior (TA) muscle was firmly tied from the tendon and the knee, dissected out, placed in a Plexiglas bath filled with oxygenated Krebs–Ringer solution, at 37 °C, and tied to a MLT 1030/D force transducer (ADinstruments, Oxford, UK). To determine isometric muscle force, the optimum muscle length and stimulation voltage were determined from micromanipulations of muscle length to produce the maximum isometric twitch force using a S48 Stimulator (Grass Research Instruments, West Warwick, RI, USA), that was controlled and measured using a PowerLab 4/35 device (AD Instruments, Oxford, UK). Isometric net force was determined with a stimulation frequency in the range 1–200 Hz for 450 ms, with 2 min of rest between stimuli. Specific net force was determined from the relationship between isometric net force (mN/mm2) using total muscle fiber cross-sectional area (mm^2^) to calculate it, as described [60]. Electromyography (EMG) was performed on isofluorane-anesthetized mice using Powerlab 26 T data acquisition hardware and LabChart 8 software (ADInstruments, Oxford, UK). Electrode distribution setup in mice was performed as described [16]. Briefly, the sciatic nerve was stimulated with two needle electrodes placed over the lumbar vertebral column. EMG was recorded by a needle electrode inserted into the right gastrocnemius muscle. The other recording electrode was subcutaneously placed in the right paw of the hindlimb. Repetitive nerve stimulation (RNS) protocol consisted in a 5 mA stimulation pulse at 50 Hz for 4-s. Finally, a single pulse of 5 mA was performed 7 s after RNS (post RNS). Compound muscle action potential (CMAP) values were calculated by the peak-to-peak amplitude values recorded. All-recorded CMAP values of the RNS stimulation were plotted to show the decreased values after a RNS or the fatigue. The first, the last and the post RNS CMAP obtained values were also separately plotted to determine the initial, fatigue and recovery values, respectively.

### Immunoblot

Samples of TA muscles, sciatic nerves, and spinal cord were homogenized in Tris-EDTA or RIPA (150 mM NaCl, 1 mM EDTA, 1% NP-40, 0.5% sodium deoxycholate, 0.1% SDS, 50 mM Tris pH 8.0) buffers containing a cocktail of protease inhibitors along with 2 mM sodium orthovanadate, 100 mM sodium fluoride, and 1 mM phenylmethylsulfonyl fluoride. For immunoblotting, 20–50 μg of total proteins were loaded in each lane and fractionated by sodium dodecyl sulphate-polyacrylamide gel electrophoresis (SDS-PAGE), transferred onto polyvinylidene difluoride (PVDF) membranes (EMD Millipore Corp.), and probed with mouse anti myosin heavy chain (MyHC) (1:1000), mouse anti troponin C (both from Developmental Studies Hybridoma Bank of the University of Iowa, USA), rabbit anti fibronectin (1:5000; Sigma-Aldrich), rabbit anti atrogin-1 (1:500), rabbit anti MuRF-1 (1:500; both from ECM Biosciences, Versailles, KY, USA), mouse anti TrkB (1:500; BD Biosciences, Franklin Lakes, NJ, USA), rabbit anti p-TrkB Y816 (1:500; Merck-Millipore, Burlington, MA, USA), rabbit anti BDNF (1:1000; Alomone Labs, Jerusalem, Israel). Mouse anti GAPDH (1:1000; Santa Cruz Biotechnology, TX, USA) or rabbit anti β-actin (1:2000; Abcam, Cambridge, UK) antibodies were used as loading controls. In control experiments of skeletal muscle atrophy, TA muscles were obtained from adult WT mice treated with angiotensin II, as described [60]. All immunoreactions were visualized by enhanced chemiluminescence (Thermo Fisher Scientific, Waltham, MA, USA) and acquired using a Fotodyne FOTO/Analyst Luminary Workstations System (Fotodyne, Inc., Walnut Ridge, WI, USA). Data are expressed as the ratio of the protein of interest v/s loading control band intensity ratios, which were quantified using the ImageJ software [60].

### Skeletal muscle staining

Feshly frozen TA muscles were embedded in optimal cutting temperature (OCT) compound (Sakura Fine technical Co., Torrance, CA), sectioned every 20 μm with a cryostat (Thermo Scientific Microm HM 525) and mounted on Vectabond (Vector Laboratories) coated slides. Muscle fiber morphology was analyzed with conventional haematoxylin/chromotrope staining. Skeletal muscle sarcolemma and nuclei were stained with 1 μg/ml Alexa488-conjugated wheat germ agglutinin (WGA, Invitrogen) and 0.3 μM DAPI (Thermo Fisher), respectively [89]. Cryosections were also stained with a NADH reduced solution (Tris-buffer, pH 7.4, NADH reduced, nitro-blue tetrazolium) (Sigma-Aldrich) for 45 min, and fibers were classified into slow (dark blue), intermediate (blue), and fast twitch (light blue). The identity and cross-sectional area (CSA) of > 100 fibers per type per mouse were determined using the ImageJ software and are expressed as the percentage of the total [89]. For collagen second harmonic generation imaging, cryosections (20 μm) of TA muscles were imaged using an inverted Zeiss LSM 780 multiphoton laser scanning confocal microscope at the CMA Bio-Bio facility (University of Concepcion, Chile). An excitation wavelength of 800 nm was used for 400 nm detection, using a 40X objective. For immunohistochemistry, cryosections (20 μm) of TA muscles were fixed with 0.5% v/v formaldehyde (Merck-Millipore) for 90 min, washed with PBS 1X containing 0.5% Triton X-100, blocked in PBS 1X, 0.5% Triton X-100 and 4% BSA for 16–18 h at 4 °C, and incubated with mouse anti myogenin (1:5), mouse anti embryonic MyHC (1:5; both from the Developmental Studies Hybridoma Bank), rabbit anti fibronectin (1:200; Sigma-Aldrich), rabbit anti p-TrkB Y816 (1:200; Merck-Millipore), and rabbit anti BDNF (1:200; Alomone Labs) antibodies for 16 h at 4 °C. After washing, samples were incubated with a Cy2-conjugated anti mouse antibody along with Alexa555-conjugated BTX, washed, and mounted with fluorescence mounting medium (DAKO). In control experiments of skeletal muscle degeneration/regeneration, the TA muscle of adult WT mice was injected along its whole length with an aqueous 1.2% mass/volume barium chloride solution, as described [7]. Samples were obtained after 10 days of recovery and subjected to immunohistochemistry. In control experiments of skeletal muscle denervation, the sciatic nerve of adult WT mice was exposed and a 3–5 mm section was resected, as described [51]. After 7 days, TA muscles were dissected and processed for immunohistochemistry, as above described.

### Anatomical and behavioral analyses

The kyphotic index (KI) was calculated from X-ray images (Veterinary Clinic, University of Concepcion) based on the distance (D1) from the seventh cervical vertebra (C7) to the sixth lumbar vertebra (L6) and the perpendicular distance (D2) from D1 to the point of maximum vertebra curvature. KI corresponds to the D1/D2 ratio and is inversely proportional to kyphosis [47, 58]. The footprint test measures stride length and the distance between fore and hind limb paw prints left on a filter paper by mice trained to run down a runway with painted feet [6]. Balance and motor coordination was evaluated using the rotarod test. Mice were positioned on horizontal cylinders with increasing acceleration from 4 to 40 rpm for 120 s and the latency time was recorded. Eight measurements were taken per mouse, with 2 min rest, on two consecutive days [6]. In the horizontal triple bar test, mice were positioned in the center of 3 horizontal rods of different diameters (2, 4 and 6 mm). The time taken by the mice to reach the end of each rod was recorded and scored, as described [14]. In the static bars test, mice were positioned at the end of 5 wooden bars of decreasing diameters (31 to 8 mm), which are joined at one end to a platform, and the time the mice take to reach the free end was recorded [14].

### Strength measurements

In the Kondziela’s inverted screen test, mice were positioned in the center of a metallic mesh which was rotated 180°. The time taken before the mouse detached from the mesh was quantified and a score assigned. Measurements were made 3 times for each mouse separated by 30 min with a maximum test duration of 120 s [15]. In the weights test, mice were challenged to hold different weights (15.5, 23.1, 30.8, 39.4, 46.4, 54.1 g) with their forelimbs while suspended from the tail. A score was assigned according to the time they were able to hold the different weights. The maximum time of maintenance score was 3 s. The final score was normalized to the body weight of each mice [15, 60]. When indicated, p75^NTR^−/− mice were subcutaneously injected daily with 3 μmol/Kg of the acetylcholinesterase inhibitor pyridostigmine bromide in commercial physiological saline [50]. Controls were similarly injected with vehicle. One hour after drug administration, mice were challenged in the weights test.

### Statistical analyses

Statistical analyses and plots were performed using the GraphPad Prism 5.0 software. The data are expressed as the mean value ± SEM. Statistical analyses of one variable were performed using the Student’s t-test of unpaired data. For the statistical analyses in which several variables were considered, the two-way-ANOVA test was used. A level of *p* < 0.05 was considered significant.

## Results

### The p75^NTR^ receptor is required for postsynaptic morphological organization and ultrastructural assembly at the NMJ

**Fig. 1:**
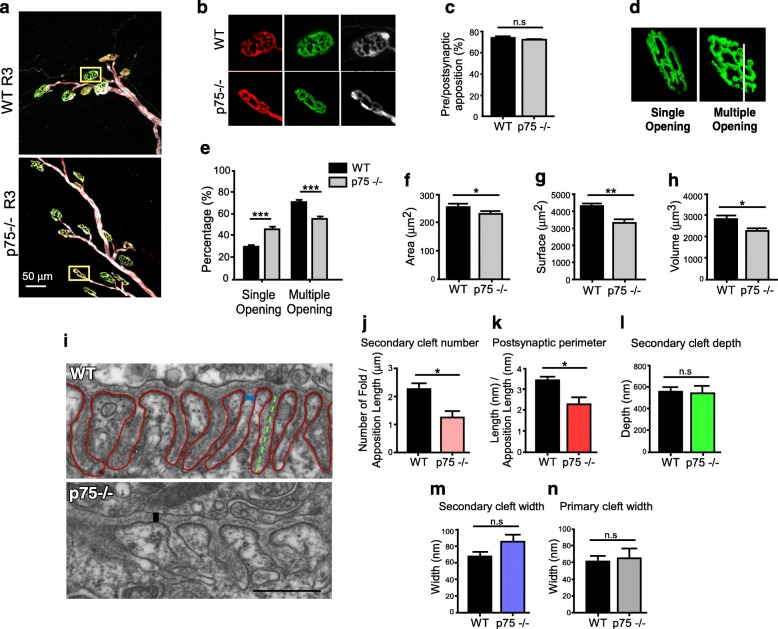
Abnormal NMJ postsynaptic complexity and structure in p75^NTR^−/− mice. **a-c** Whole-mounts of LAL muscles of 2–4 months old WT and p75^NTR^−/− mice were stained to reveal presynaptic motor terminals (2H3 plus SV2 antibodies, red), postsynaptic AChRs (BTX, green) and terminal Schwann cells (S-100 antibody, white). Laser confocal images of the R3 innervation region of WT and p75^NTR^−/− mice LAL muscle (**a**) show full coverage of AChR pretzels by presynaptic motor axons and terminal Schwann cells (**b**). Scale bar: 50 μm. **c** Quantification of the apposition of postsynaptic AChR pretzels by presynaptic motor axons. The plots correspond to > 35 NMJs per mice. **d**-**h** Diaphragm muscles from 2 months old WT and p75^NTR^−/− mice were stained with fluorescent-conjugated BTX to reveal AChR aggregates. **d** Postsynaptic apparatuses were classified into those having one or multiple peripheral openings and the relative abundance of these shapes was quantified (**e**). The plot corresponds to 130–220 NMJs per mouse. From 3D images, the area (**f**), surface (**g**) and volume (**h**) of individual pretzels were quantified. The plots correspond to 50–100 NMJs per animal. **i**–**n** Diaphragm NMJs from 2 to 4 months old p75^NTR^−/− and WT mice were analyzed by transmission electron microscopy. **i** Representative images of WT and p75^NTR^−/− NMJs. Scale bar: 500 nm. **j** The number of secondary folds is also expressed regarding the pre- and postsynaptic apposition length. **k** The postsynaptic perimeter was quantified as the length of the muscle membrane within the secondary folds (red line in **i**) expressed regarding the apposition length between the pre- and postsynaptic contact zone. **l** The depth of secondary folds was measured in a straight line traced between the beginnings of the invagination to the deepest point of the fold (green dotted line in **i**). **m** Quantification of the width of the secondary fold (blue line in **i**). **n** Quantification of the primary fold width, corresponding to the synaptic cleft width (black line in **i**). The plots correspond to 3–4 NMJs per animal. The bars represent the mean ± SEM of *n* = 3 (WT and p75^NTR^−/−) mice in (**a**–**c**) and (**i**–**n**), *n* = 8 (WT and p75^NTR^−/−) mice in (**d**–**h**). n.s, non-significant, *p < 0.5, **p < 0.01, ***p < 0.001, two-way ANOVA (**e**), or unpaired t-test (**c**, **f**–**h**, **j**–**n**)

As a first attempt to examine whether p75^NTR^ is involved in the structural organization of the adult NMJ, cranial LAL muscles from WT and p75^NTR^−/− mice were stained to reveal the pre and postsynaptic profiles, as well as Schwann cells. Low magnification analyses indicate no gross differences in NMJ organization, as evidenced by unaltered distribution of the main innervation profiles [61] (Additional file 1: Figure S1). Higher magnification images revealed that nerve terminal branches aligned precisely with their respective postsynaptic specialization in both WT and p75^NTR^−/− mice muscles (Fig. 1a, b). Quantitative analyses confirmed that NMJs are fully innervated, as the area of presynaptic staining apposes more than 70% of the postsynaptic domain in both genotypes [40] (Fig. 1c). In addition, myonuclei from TA muscle fibers from both genotypes do not express detectable levels of myogenin (Additional file 2: Figure S2a), a molecular marker of muscle denervation [43]. Similarly, terminal Schwann cells distribute in precise register with presynaptic terminals and postsynaptic AChR staining in p75^NTR^−/− and control NMJs (Fig. 1b). Despite these results, we consistently found that postsynaptic domains of p75^NTR^−/− mice were smaller and less complex than those from WT mice (Fig. 1b). As postsynaptic maturation is characterized by the presence of AChR-poor regions [54], we quantified postsynaptic structures containing a single (i.e. still maturing) or multiple peripheral openings (i.e. fully mature) (Fig. 1d). NMJs from 2-month-old p75^NTR^−/− mice show a significant decrease in the proportion of mature pretzel-like shapes, compared to WT controls (Fig. 1e). In 3D projections of NMJs from both genotypes (Additional file 3: Figure S3), we quantified significantly decreased values of postsynaptic area (Fig. 1f), surface (Fig. 1g) and volume (Fig. 1h), consistent with the relative abundance of less complex maturing structures found in p75^NTR^−/− mice. Based on these findings, we next analyzed potential postsynaptic NMJ defects at the ultrastructural level (Fig. 1i). Whereas the secondary clefts of control NMJs are long, thin, and closely spaced, these structures are reduced in number (Fig. 1j) and give rise to decreased postsynaptic perimeter in p75^NTR^−/− mice (Fig. 1k). Our quantitative analyses revealed that other parameters, such as the depth and width of the secondary folds, as well as the width of the primary fold, are not significantly affected in p75^NTR^−/− mice (Fig. 1l–n). Together, our findings reveal that the absence of p75^NTR^ negatively affects the organization of the NMJ postsynaptic domain.

### p75^NTR^ null mice muscles display increased fatigue, decreased force production and histological alterations

**Fig. 2:**
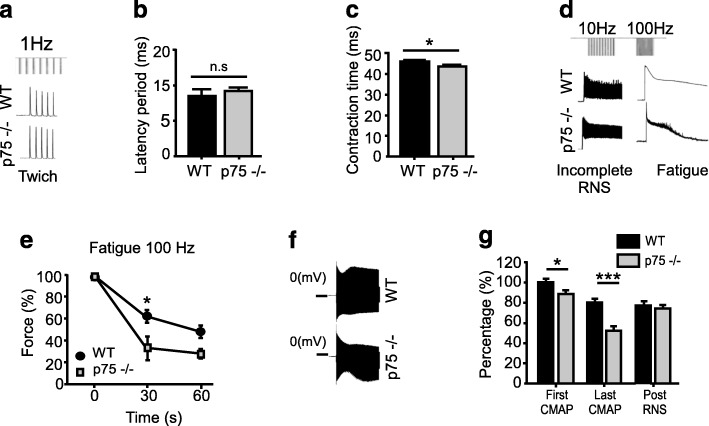
The p75^NTR^−/− mice display NMJ transmission alterations and accelerated nerve-dependent muscle fatigue. Time course of the mean force measurements (as a percentage of maximal force) for hind limb muscles after sciatic nerve stimulation of 2–4 months old WT and p75^NTR^−/− mice. First, the phases of contractile response elicited by 1 Hz sciatic nerve stimulation were measured. **a** Representative traces of 1 Hz stimulation protocol. No changes were observed in the latency period (**b**) of single-twitch stimulations, whereas a slight but significant decrease was observed in the contraction time (**c**) in p75^NTR^−/− muscles compared to WT controls. Second, force decline was determined after incomplete (10 Hz) and complete (100 Hz) tetanus. **d** Representative traces of 10 and 100 Hz stimulation protocols. A significant acceleration of muscle fatigue was observed at 30s of 100 Hz stimulation in p75^NTR^−/− compared to WT mice (**e**). **f** Representative traces of electromyographic recording after repetitive nerve stimulation. **g** Quantification of CMAP shows that p75^NTR^−/− mice display decreased impaired neuromuscular activity after the first and after repetitive presynaptic stimulation (RNS). The results represent the mean ± SEM of *n* = 3–4 (WT), *n* = 4–7 (p75^NTR^−/−) in (**a**–**e**) and *n* = 7 (WT), *n* = 8 (p75^NTR^−/−) in (**f**–**g**). n.s., non-significant, *p < 0.5, ***p < 0.001, unpaired t-test (**b**–**c**, **g**), or two-way ANOVA (**e**)

We next aimed to correlate our morphological observations with functional NMJ parameters by analyzing the effect of sciatic nerve stimulation on hind limb muscle force generation and fatigue. After single twitch stimulation (Fig. 2a) we found no differences in the latency period (Fig. 2b) and a slight but significant decrease in the contraction time in p75^NTR^−/− mice (Fig. 2c). After repetitive nerve stimulation protocols (Fig. 2d), we found that p75^NTR^−/− mice muscles displayed a significantly increased fatigue after 30s of tetanic 100 Hz nerve stimulation (Fig. 2e). We complemented these studies with electromyography recording after presynaptic stimulation (Fig. 2f). We found that p75^NTR^−/− mice display decreased CMAP, a feature that was enhanced after repetitive nerve stimulation (Fig. 2g). After 7 s of repetitive nerve stimulation, single CMAP values were similar in control and p75^NTR^−/− mice muscles (Fig. 2g). Together, these results reveal that p75^NTR^−/− mice display altered nerve-induced muscle responses.

**Fig. 3:**
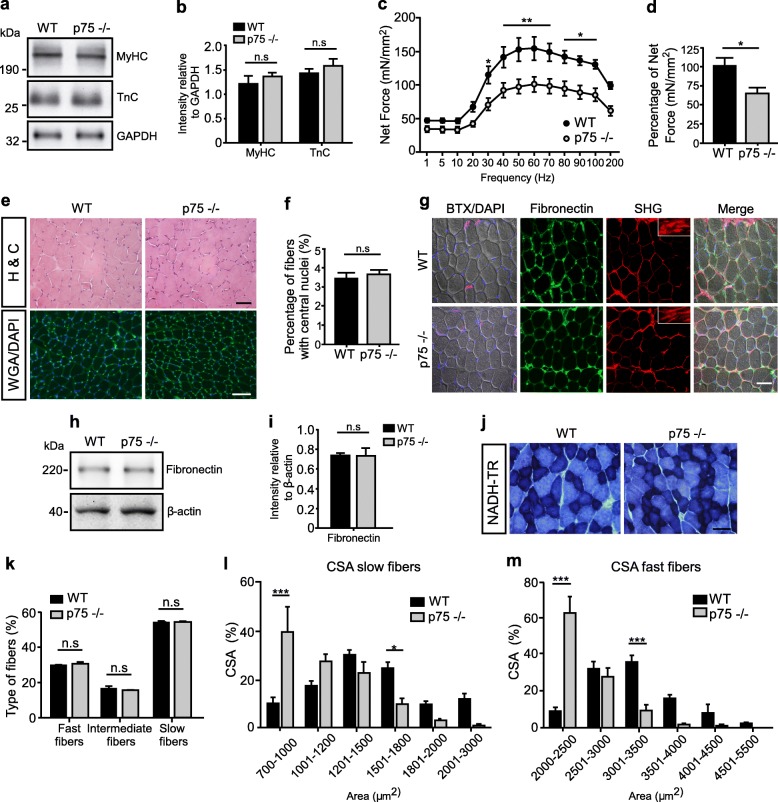
Skeletal muscle properties of p75^NTR^−/− mice. **a** Total protein samples of Gastrocnemius muscles from 2 to 4 months old WT and p75^NTR^−/− (*n* = 4) mice were analyzed by Western blot using specific antibodies to detect myosin heavy chain (MyHC) and Troponin C (TnC). The levels of GAPDH were used as loading control. **b** Quantification of the relative levels of the sarcomeric proteins was performed by band intensity densitometry and expressed as a ratio of GAPDH band intensity. **c** Contraction force curve as a function of stimulation frequency (Hz) in isolated TA muscle. **d** Maximal isometric force was determined in WT (*n* = 6) and p75^NTR^−/− (*n* = 4) mice. **e** Cross-sections of TA muscles from 2-months-old WT and p75−/− mice were evaluated by hematoxylin/chromotrope staining (H&C) and WGA plus DAPI staining. Bar: 50 μm. **f** The percentage of myofibers bearing central nuclei was quantified in WGA/DAPI-stained cryosections of TA muscles from WT (*n* = 8) and p75^NTR^−/− (*n* = 8) mice. **g** Cross-sections of TA muscles from both genotypes were stained to analyze inter-fiber protein deposition. Bright field images (*fist column*) display DAPI nuclear (blue) plus BTX NMJ (magenta) staining. The same sections were processed for immunohistochemical detection of fibronectin (*second column*), as well as for the two-photon laser-based second harmonic generation technique (*third column*, SHG) to detect collagen fibers. The insets show high magnification images at the perimysium. The fourth column show the merged images. Bar: 50 μm. **h** Total protein samples of TA muscles from WT and p75^NTR^−/− (*n* = 3) mice were analyzed by Western blot using a specific antibody to detect fibronectin. The levels of β-actin were used as loading control. **i** Quantification of the relative levels of fibronectin was performed by band intensity densitometry and expressed as a ratio of β-actin band intensity. **j** Cross-sections of TA muscles from both genotypes were histochemically stained to detect NADH-TR activity. Scale bar: 50 μm. **k** Quantification of fiber types in at least 400 fibers per WT or p75^NTR^−/− mice. Results are expressed as a percentage of the total quantified fibers in a central region of interest. **l** Distribution histogram of the cross-sectional area (CSA) of slow-twitch fibers. **m** Distribution histogram of the CSA of fast-twitch fibers. The results represent the mean ± SEM of *n* = 8 (WT and p75^NTR^−/−) mice by quantifying at least 100 fibers per mice. n.s., non-significant, *p < 0.5; **p < 0.1; ***p < 0.01, unpaired t-test (**b**, **d**, **f**, **i**) or two-way ANOVA (**c**, **k**–**m**)

To analyze if sustained defective synaptic activity resulted in skeletal muscle defects in p75^NTR^−/− mice, we first quantified the total levels of the sarcomeric proteins myosin heavy chain (MyHC) and troponin C (TnC) by Western blot (Fig. 3a). Compared to the loading control GAPDH, we found no differences in the levels of both proteins in the different genotypes (Fig. 3b). Next, we measured the force elicited after direct stimulation of the TA muscle at different frequencies. Our findings show a significant decrease in the net isometric force in the range of 30 to 100 Hz stimulation in p75^NTR^−/− TA muscles (Fig. 3c). These findings correlate with a significant reduction in the percentage of maximum isometric contraction force developed by p75^NTR^−/− mice muscles after 60 Hz stimulation (Fig. 3d). As these findings reveal defective muscle contraction in p75^NTR^−/− mice, we next studied muscle structure by histological analyses of transversal TA muscle cryosections (Fig. 3e). Our results show no gross differences in muscle fiber distribution or mononuclear infiltration (H&C staining). We also observed no differences in spontaneous muscle fiber degeneration/regeneration cycles, as cryosections from both genotypes show similar WGA/DAPI staining (Fig. 3e), a similarly low proportion of myofibers displaying central nuclei (Fig. 3f), and the absence of fibers expressing an embryonic form of MyHC (Additional file 2: Figure S2b), a molecular marker of muscle regeneration [32]. To evaluate potential differences in inter-fiber extracellular matrix deposition, we first performed immunohistochemical staining (Fig. 3g) and Western blot analyses (Fig. 3h, i) to study the levels of fibronectin. We found no differences in fibronectin levels or distribution in TA muscle samples from both genotypes. To complement these studies, we standardized the second harmonic generation technique, a two-photon laser confocal analysis that allows label-free imaging of collagen fibers at high resolution on native tissue [23]. Following this approach, we consistently found that the levels of collagen deposition, as well as the normally linear organization of collagen fibers, are not affected in skeletal muscles of the p75^NTR^−/− mice, compared to controls (Fig. 3g). We next evaluated muscle fiber plasticity by histochemical staining to reveal NADH-thioreductase (NADH-TR) activity (Fig. 3j). Quantification shows no differences in the relative composition of p75^NTR^−/− and control TA muscles regarding fast (light blue), intermediate (blue), and slow-twitch (dark blue) fibers (Fig. 3k). However, we found a significant reduction in the cross-sectional area (CSA) of muscle fibers of p75^NTR^−/− mice, evidenced by a strong increase in the proportion of low caliber fibers and a corresponding decrease in higher caliber fibers of both, slow and fast-twitch muscle fibers (Fig. 3l–m). To analyze if the decrease in muscle fiber size observed in p75^NTR^−/− mice was due to atrophy, we conducted Western blot experiments to analyze the levels of MuRF-1 and atrogin-1, two E3 ubiquitin ligases that act as key regulators of ubiquitin-mediated protein degradation in skeletal muscle [4]. We detected similar low levels of both proteins in TA muscle samples from both, control and p75^NTR^−/− mice (Additional file 2: Figure S2c). Together, these findings evidence that the absence of p75^NTR^ results in functional NMJ defects, which correlate with altered structure and contractile capabilities of skeletal muscles.

### p75^NTR^ null mice display abnormal motor behavior

**Fig. 4:**
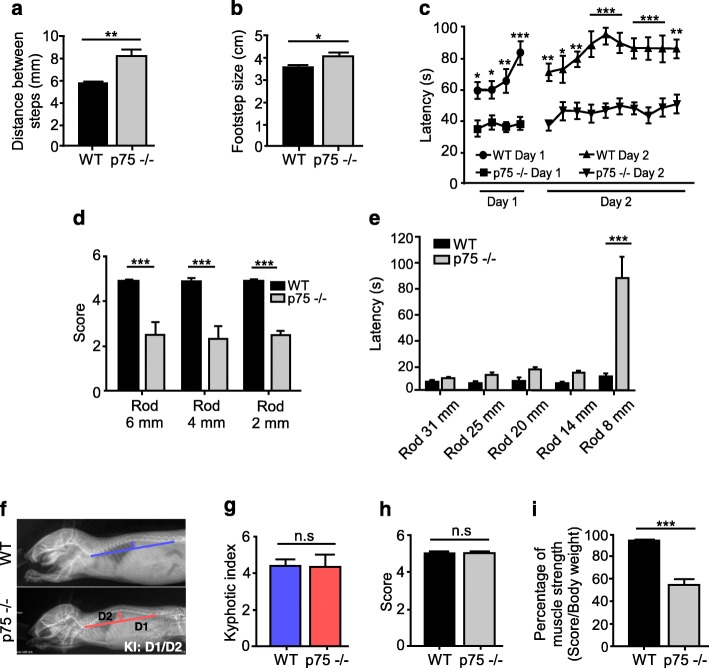
Altered motor performance and muscle strength in p75^NTR^−/− mice. **a**–**e** Young (2–4 months old) WT (*n* = 8) and p75^NTR^−/− (*n* = 8) mice were challenged in different motor tests. In the footprint test, p75^NTR^−/− mice displayed increased distance between steps (**a**) and increased distance between the fore and hind limbs steps (**b**) compared to WT controls. In the accelerating rotarod test, the latency time in two consecutive days was significantly decreased in p75^NTR^−/− mice compared to WT controls (**c**). Similarly, in the triple horizontal bar test (**d**) and the static bar test (**e**) p75^NTR^−/− mice had reduced scores and latency, respectively, compared to WT mice. **f** Representative X-ray images of WT and p75^NTR^−/− mice. The kyphotic index (KI) was calculated as the ratio between distances D1 and D2. **g** KI quantification show no differences between WT (*n* = 4) and p75^NTR^−/− (*n* = 3) mice. **h**–**i** Young (2–4 months old) WT and p75^NTR^−/− mice were challenged in two muscle strength tests. **h** In the Kondziela’s inverted screen test, no differences were detected between WT and p75^NTR^−/− mice. However, in the weights test (**i**), p75^NTR^−/− mice (*n* = 6) obtained a significantly impaired score compared to WT mice (*n* = 5). The results represent the mean ± SEM. n.s., non-significant, *p < 0.5; **p < 0.1; ***p < 0.01, unpaired t-test (**a**, **b**, **g**–**i**), or two-way ANOVA (**c**–**e**)

To study if the alterations in muscle contraction, ultrastructure, and function of the neuromuscular synapse found in p75^NTR^−/− mice resulted in defective motor behavior, several motor coordination and balance tests were performed. We first found that p75^NTR^−/− mice display altered walking gait, evidenced by an increase in the step distance (Fig. 4a) and in the traces between fore and hind limbs (Fig. 4b). Also, p75^NTR^−/− mice displayed a shorter latency time to fall in the rotarod test (Fig. 4c), and required more time to reach the end, or fell out, either in the triple horizontal bars (Fig. 4d) or in the static bar tests (Fig. 4e). As motor alterations are due to anatomical defects in mice models displaying NMJ phenotypes [58], we also measured the kyphotic index (i.e. abnormal spine curvature); however, no differences were found between p75^NTR^−/− and WT mice (Fig. 4f–g). Based on our previous findings, we next evaluated whether the absence of p75^NTR^ results in force impairment in the context of the entire animal. Our results show that p75^NTR^−/− mice are able to hold their body weights in the inverted grid test, as WT mice do (Fig. 4h). However, when challenged to hold increasing weights with their fore limbs, p75^NTR^−/− mice show a significantly impaired performance (Fig. 4i). Thus, the absence of p75^NTR^ results in defective locomotor behavior.

### The absence of p75^NTR^ affects the ultrastructure of motor nerve terminals

**Fig. 5:**
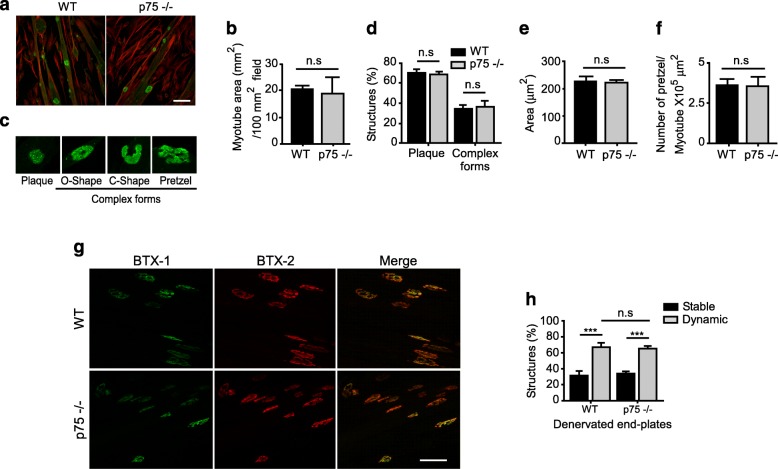
Muscle-derived p75^NTR^ is not required for nerve-independent AChR aggregation. Myoblasts from the gastrocnemius muscle of 2–4 months old WT and p75^NTR^−/− mice were induced to fuse into myotubes for 5 days onto poly-ornithine/laminin coated dishes. **a** Representative laser confocal images show myotubes from both genotypes stained to reveal actin (phalloidin, red) and AChR aggregates (BTX, green) Scale bar: 50 μm. **b** Quantification of myotube area per field show no differences amongst both genotypes. **c** Morphology of aneurally-induced complex AChR aggregates, which were classified into “plaques” and “complex shapes”, the latter including O-, C-, and pretzel-like shapes. **d** The relative abundance of AChR shapes was quantified and expressed as a fraction of the total AChR structures. **e** The area of AChR shapes was also determined. **f** Quantification of the number of AChR shapes expressed regarding the myotube area. The plots represent the mean ± SEM of n = 4 (WT and p75^NTR^−/−) mice. Three cultures for each animal and 30–50 AChR aggregates were analyzed per experiment. **g**–**h** For the in vivo two-color BTX method, LAL muscles from WT and p75^NTR^−/− mice were labeled with BTX-1 and the right hemi-LAL muscle was subsequently denervated by facial nerve transection. After a 7-day recovery period, LAL muscles were dissected and labeled with BTX-2. NMJs in maximum intensity projection images of LAL whole mounts (**g**; Bar: 50 μm) were categorized in “stable” or “dynamic” according to both BTX relative intensities, and quantified (**h**). n.s., non-significant, ***p < 0.01, unpaired t-test (**b**, **e**, **f**), or two-way ANOVA (**d**, **h**)

Mature NMJs express low levels of p75^NTR^ in motor axon terminals, terminal Schwann cells, and muscle fibers, in close vicinity to AChR aggregates [26, 27]. In order to gain insights on the cellular source of the p75^NTR^ fraction potentially involved in postsynaptic NMJ organization and ultrastructural assembly, we first analyzed the aneural formation of complex AChR structures on cultured myotubes obtained from satellite cells of WT and p75^NTR^−/− mice (Fig. 5a). Our analyses revealed similar myotube formation and size in both genotypes (Fig. 5b). When seeded onto polyornithine/laminin matrices, myotubes assemble complex postsynaptic structures resembling those observed in vivo [45]. We categorized AChR aggregates into “immature plaques”, having homogenous AChR distribution, and “complex maturing forms”, corresponding to those having AChR-poor subregions forming “O-”, “C-” or “pretzel-like” shapes (Fig. 5c). Our morphological classification indicates no differences in the complexity of AChR aggregates amongst myotubes derived from p75^NTR^−/− and WT mice (Fig. 5d). Consistently, no differences were detected in the size of postsynaptic structures (Fig. 5e) or in the ability of myotubes from both genotypes to assemble complex postsynaptic structures, quantified as the number of AChR complex structures per myotube area (Fig. 5f). These findings suggest that the absence of the muscle-derived p75^NTR^ receptor does not alter the assembly of mature postsynaptic structures in the p75^NTR^−/− mice in vivo.

As a way to investigate the possibility that muscle-derived p75^NTR^ affects postsynaptic maintenance, the dynamics of AChRs after NMJ denervation were analyzed following an in vivo two-color BTX method [29]. With this aim, NMJ postsynaptic domains of LAL muscles were labeled with a non-saturating dose of a fluorescently tagged BTX (BTX-1) in live mice from both phenotypes and the right hemi-LAL muscle was subsequently subjected to denervation through facial nerve resection. After a 7-day recovery period, the pre-labeled muscles were dissected and labeled with a different fluorescently tagged variant of BTX (BTX-2). Using confocal microscopy, AChR aggregates were categorized in “stable” if BTX-1 and BTX-2 labels were similarly intense, or as “dynamic” if BTX-1 labelling was mostly absent and BTX-2 intensity was comparatively higher (Fig. 5g). As expected, denervation resulted in a significant increase in the proportion of dynamic AChR aggregates in LAL muscles from both, control and p75^NTR^−/− mice; however, no differences were detected in the proportion of dynamic AChR aggregates amongst denervated LAL muscles from p75^NTR^−/− and WT mice (Fig. 5h). These findings suggest that the absence of the muscle-derived p75^NTR^ does not differentially affect the stability of mature postsynaptic structures after denervation.

**Fig. 6:**
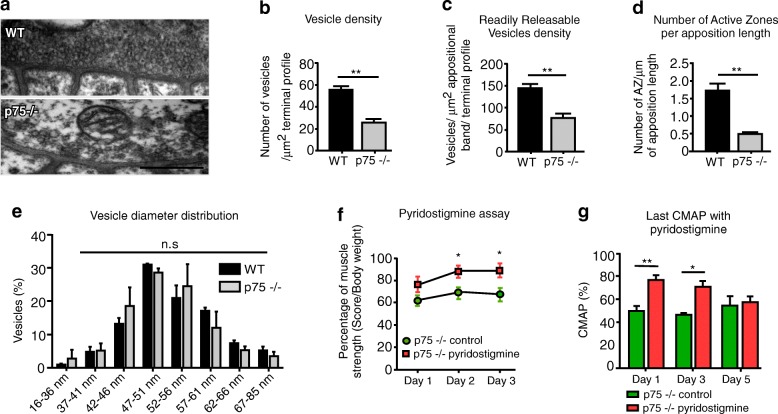
Reduced presynaptic vesicles in p75^NTR^−/− mice contribute to impaired synaptic transmission and muscle strength. **a**–**e** Diaphragm NMJs from 2 to 4 months old WT and p75^NTR^−/− (*n* = 3) mice were analyzed by transmission electron microscopy. **a** Representative images of WT and p75^NTR^−/− NMJs. Scale bar: 500 nm. **b** The total number of vesicles in each axon terminal was quantified as a fraction of the synaptic terminal area. **c** To quantify the RRP of vesicles, the number of vesicles within a 480 nm strip directly across active zones was counted. **d** Active zones (AZ) were quantified as electro dense regions located in direct apposition to postsynaptic secondary folds. The results are expressed as a function of the length of pre/postsynaptic apposition. **e** Vesicle diameter was measured in 40–110 vesicles per nerve terminal (*n* = 3–4 NMJs per mice). **f**–**g** Pharmacological inhibition of acetylcholinesterase with daily subcutaneous administration of pyridostigmine bromide partially rescued muscle strength (*n* = 5) (**f**) and NMJ transmission (*n* = 4) (**g**) of p75^NTR^−/− mice. The results represent the mean ± SEM. n.s., non-significant, *p < 0.5; **p < 0.01, unpaired t-test. **b**–**d**, **f**, **g**, or two-way ANOVA (**e**)

Even though we did not find differences in the profile of muscle fiber innervation (Fig. 1a–c), our previous findings showing functional NMJ defects in p75^NTR^−/− mice prompted us to examine the presynaptic ultrastructure (Fig. 6a). Remarkably, quantitative analyses showed a drastic decrease in the total vesicle density in p75^NTR^−/− motor terminals (Fig. 6b). p75^NTR^−/− mice also displayed a significant reduction in the density of the readily releasable pool of vesicles (RRP) (Fig. 6c), as well as a strong decrease in the number of active zones per unit length of terminal apposition (Fig. 6d). No differences were found in the diameter of individual synaptic vesicles (Fig. 6e).

Considering that p75^NTR^−/− mice display decreased number of vesicles in the motor axon terminal as well as postsynaptic morphological defects, we hypothesized that impaired neurotransmission could contribute to deficient NMJ function, subsequent muscle contraction impairment, and defective locomotor performance. To experimentally address this idea, pharmacological interventions were conducted in which the enzyme acetylcholinesterase was inhibited by subcutaneous administration of pyridostigmine bromide. After daily treatment with the drug, p75^NTR^−/− mice were challenged in the weights test to evaluate muscle strength. Our results show that p75^NTR^−/− mice had partially rescued strength at 2 and 3 days after daily drug administration compared to p75^NTR^−/− mice treated with physiological saline (Fig. 6f). Acetylcholinesterase inhibition also improved the electromyographic recording of p75^NTR^−/− NMJs, reflected in increased CMAP activity of p75^NTR^−/− pyridostigmine treated animals from 1 to 3 days (Fig. 6g). Our results also showed that the positive effects of acetylcholinesterase inhibition are not sustained in time, as CMAP values did not increase after 5 days of treatment (Fig. 6g). Altogether, these findings reinforce the idea that impaired neurotransmission contributes to deficient NMJ function in p75^NTR^−/− mice.

### NMJs of p75^NTR^ null mice display unaltered distribution of BDNF and TrkB

**Fig. 7:**
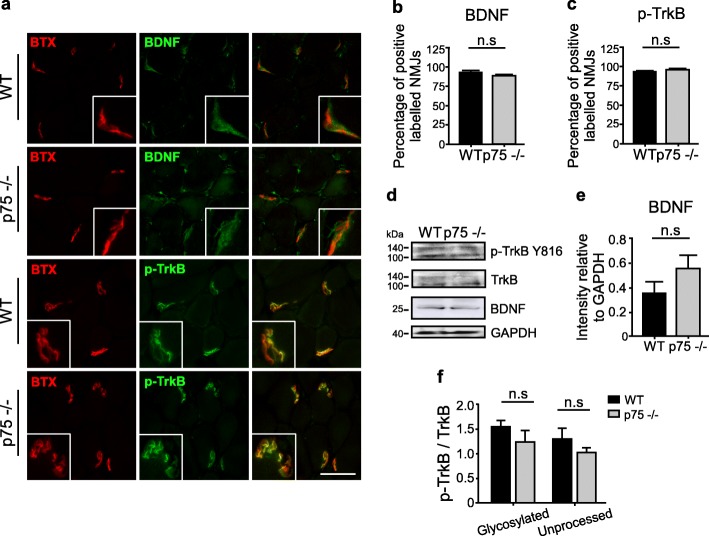
Unaltered BDNF/TrkB signaling at the NMJ of p75^NTR^−/− mice. **a** TA muscle cryosections from WT and p75^NTR^−/− mice were double-labeled with antibodies (green) to detect p-TrkB Y816 or BDNF, along with BTX (red). **b**–**c** Quantification of the percentage of NMJs labeled with BNDF (**b**) or with p-TrkB Y816 (**c**). **d** Total protein samples of TA muscles from WT and p75^NTR^−/− (*n* = 3) mice were analyzed by Western blot using specific antibodies to detect p-TrkB Y816, total TrkB, and BDNF. The levels of GAPDH were used as loading control. **e**–**f** BDNF/TrkB signaling was estimated by band intensity densitometry of BDNF (**e**) and by the Y816 phosphorylation of both, the glycosylated (145 kDa) and the unprocessed (110 kDa) forms of TrkB and expressed as a ratio of the corresponding bands detected with an anti TrkB antibody (**f**). n.s., non-significant, unpaired t-test

It has been demonstrated that the levels of NTs and their Trk receptors are altered in conditions affecting the neuromuscular synapse. For instance, hind limb muscles of amyotrophic lateral sclerosis mouse models display a significant decline in the number of NMJs expressing BDNF, NT-4, GDNF, p75^NTR^, TrkB, and TrkC [31]. Therefore, as a first hint to analyze the potential involvement of NT-dependent signaling on the morphological and functional alterations observed in p75^NTR^−/− mice, we analyzed the levels of expression of BDNF and its TrkB receptor at the NMJ, as BDNF/TrkB signaling plays important roles on NMJ organization, including synaptic vesicle availability [34, 75]. With that aim, cryosections of TA muscles from control and p75^NTR^−/− mice were subjected to immunohistochemistry to detect BDNF and an active form of TrkB (p-TrkB Y816) (Fig. 7). Our findings show that NMJs from control and p75^NTR^−/− mice display positive staining for BDNF and p-TrkB Y816 (Fig. 7a). Immunofluorescence quantification shows that the vast majority of NMJs express both proteins in TA muscle cryosections of both genotypes (Fig. 7b, c). Following Western blot experiments, we analyzed the levels of TrkB and BDNF. We also included the antibody that specifically detects active TrkB when phosphorylated at residue Y816 of both, the glycosylated (145 kDa) and the unprocessed (110 kDa) forms of TrkB [38]. Our results show that the total levels of BDNF and TrkB are not significantly altered in p75^NTR^−/− mice muscles (Fig. 7d). Consistently, immunoblot quantifications show that the levels of pro-BDNF (the precursor form of BDNF) or the active p-TrkB Y816 form of TrkB were unchanged by the absence of p75^NTR^ at the NMJ (Fig. 7e, f). Similarly, the levels and distribution of BDNF and TrkB in samples of sciatic nerve and the spinal cord of p75^NTR^−/− mice were similar to control mice (Additional file 4: Figure S4). Together, these findings suggest that BDNF/TrkB signaling is not significantly altered at the neuromuscular synapse of p75^NTR^−/− null mice.

## Discussion

Dysfunctions of the NMJ are caused by traumatic spinal cord or peripheral nerve injuries as well as by severe motor pathologies [35, 36, 59]. Despite the remarkable regenerating ability of the peripheral nervous system, delayed NMJ regeneration paradigms show that, even though muscles are reached by motor axons and re-build morphologically normal NMJs after long-term denervation, regeneration after a critical period is not associated with a positive functional outcome in distal muscles [53, 70], suggesting synaptic rather than regenerative failures after critical periods of time. In mice models of amyotrophic lateral sclerosis, it has been demonstrated that NMJ disruption precedes subsequent motor neuron death [59], demonstrating a critical primary role for NMJ maintenance in the etiology of this neurodegenerative disease. Together, these findings reveal that cells at the damaged NMJ niche express signals that impair synaptic maintenance and repair [78]. Cumulative evidence shows that p75^NTR^ is likely one such molecule. Although Schwann cell-derived p75^NTR^ plays a major role on the perinatal elimination of muscle fiber poly-innervation [37, 92], NMJs of P14 p75^NTR^−/− mice showed no evident alterations regarding AChR clustering and their innervation profile [37], suggesting a minor function for this receptor on the maintenance and function of mature NMJs. Remarkably, various experimental paradigms of nerve injury result in strongly increased p75^NTR^ expression in motor neurons [19, 42, 69] and Schwann cells [30, 65, 79]. Also, p75^NTR^ expression is up-regulated in spinal cord motor neurons in mice models of amyotrophic lateral sclerosis [41, 52, 72]. Rather than a positive outcome given by its role as a receptor for NTs, cumulative evidence has demonstrated that p75^NTR^ up-regulation impairs nervous system repair, a feature related to its additional role as a cell death mediator in various neuronal and glial populations [1, 8, 20–22]. Indeed, p75^NTR^ inhibition has been successfully tested as a pharmacological target to delay disease progression [35, 74], and chronic administration of a p75^NTR^ antisense peptide nucleic acid [81] or a p75^NTR^-derived trophic cell-permeable peptide delays motor dysfunction and mortality in amyotrophic lateral sclerosis mice models [56]. Despite this evidence, the outcome of chronic p75^NTR^ inhibition on the maintenance of mature NMJs has not been studied. Therefore, we aimed to deeply characterize, using neuroanatomical and neurophysiological tools, the structure and function of p75^NTR^−/− mice NMJs. Our finding show that the absence of p75^NTR^ impairs postsynaptic organization and ultrastructural complexity of the NMJ, which correlate with altered synaptic function at the levels of nerve activity-induced muscle responses, muscle fiber structure, force production, and locomotor performance.

Our studies provide the first evidence that chronic p75^NTR^ deficiency results in aberrant NMJ maturation accompanied by altered pre- and post-synaptic structure and defective neurotransmission. We found that the absence of p75^NTR^ results in a significant reduction in the number of synaptic vesicles and active zones, as well as in the density of the RRP of vesicles, supporting the idea that a reduction in ACh release could account for the observed motor phenotypes in p75^NTR^−/− mice. Our results also reveal defects in postsynaptic complexity at the NMJ of p75−/− mice, evidenced by a reduced number of secondary folds, i.e. the membrane postsynaptic structures that increase the postsynaptic area and concentrate both, AChRs and the voltage-gated sodium channel Nav1.4, thus favoring action potential generation for subsequent muscle contraction [76, 77, 94]. As a morphological support of the defects observed in both, pre and postsynaptic domains, the use of an AChE inhibitor drug resulted in the recovery of NMJ synaptic transmission and muscle strength in p75^NTR^−/− mice. Consistent with our findings, presynaptic motor terminals are required for postsynaptic maturation, as muscle denervation at P10 halts subsequent postsynaptic plaque-to-pretzel transition [54]. Our results also show a significant alteration in motor coordination and balance in p75^NTR^−/− mice, as previously reported [64, 68, 96]. In this context, mice null for p75^NTR^ in the cerebellar external granular layer (EGL) also display altered motor performance [96]. Additionally, it has been shown that the absence of p75^NTR^ in hippocampal neurons reduces neurogenesis, resulting in some behavioral alterations but, interestingly, not in locomotor defects [9, 12]. Our studies provide novel evidence showing that, in addition to its effects on the central motor coordination, the absence of p75^NTR^ specifically alters NMJ pre- and postsynaptic organization, a feature that can at least partially explain the observed defects in muscle structure and locomotor performance.

The mouse model used throughout our studies express a p75^NTR^ isoform lacking cysteine repeats 2, 3, and 4 and express a truncated form of p75^NTR^ receptor that could potentially contribute to the phenotypes that we and others have described in these mice [86]. However, polypeptide fragments derived from this truncated form of p75^NTR^ are expressed at low levels, as they are susceptible to degradation by the α- and γ-secretases, as well as by the proteasome system; indeed, Trk-dependent signaling activates the α-secretase processing and endosomal targeting of these p75^NTR^-derived fragments [83]. If any, the p75^NTR^ protein fragments expressed by p75^NTR^ exon III null mice could play a beneficial effect in the context of the NMJ, as they have been shown to increase BDNF-TrkB dependent survival of motor neurons in vitro and in vivo in the hSODG93A mice model of amyotrophic lateral sclerosis [56]. Therefore, future experiments using complete p75^NTR^ null models will complement our observations regarding the critical effects that p75^NTR^ inhibition exerts in the organization of mature NMJs.

Our functional studies showed that repetitive presynaptic stimulation resulted in increased muscle fatigability, as well as in impaired muscle force generation and CMAP values in p75^NTR^−/− mice. Interestingly, we observed a rescue of a single CMAP value in p75^NTR^−/− mice 7 s after repetitive nerve stimulation. A comparative protocol is commonly used in the clinic to discriminate the pre or postsynaptic etiology of myasthenic diseases; CMAP values at rest are reduced but display a strong rescue 10 s after maximal voluntary contraction (MVC) in myasthenic diseases of presynaptic origin (such as the Lambert-Eaton myasthenic syndrome) due to post-activation facilitation (PAF) [49]. Also, while acetylcholinesterase inhibition increased CMAP values at days 1 and 3, this effect was lost at day 5, a finding consistent with the observation that sustained administration of cholinesterase inhibitors as a monotherapy are minimal and not sustained in time in presynaptic myasthenic diseases [84]. Therefore, despite the limitations of our approach to be compared to the clinical practice, and although a postsynaptic effect cannot be discarded, we believe that our electromyographic recordings, along with the transmission electron microscopy studies, strongly suggest a main presynaptic defect at the NMJ of p75^NTR^−/− mice. We speculate that this sustained upstream presynaptic defect impairs neurotransmitter availability, which is manifested in downstream defects in NMJ morphology, muscle fiber structure, and locomotor performance.

p75^NTR^ is a multifaceted receptor with the ability to signal through NT-dependent and independent pathways. Even though our results suggest that BDNF/TrkB signaling is not significantly affected at the NMJ of p75^NTR^−/− mice, several lines of evidence relate NT signaling with the defects we found in these mice. For instance, acute antibody-dependent blocking of p75^NTR^ impairs ACh release in immature and mature neuromuscular synapses via a BDNF/TrkB-dependent mechanisms that regulates the phosphorylation of presynaptic proteins [24, 27, 62, 75]. Our findings showing that the availability of synaptic vesicles is severely compromised in the absence of p75^NTR^ are also related to NT signaling. Indeed, decreased expression of integral synaptic vesicle proteins has been reported in cultures of neurons derived from conditions with altered NT-dependent signaling, such as TrkB, TrkC and BDNF deficient mice [18, 55, 67, 80]. In addition, NTs have been reported to be involved in synaptic vesicle fusion to the plasma membrane in nerve terminals of central synapses [55, 67, 80, 82]. In frog nerve-muscle co-cultures, the expression of synapsin I, a presynaptic protein that distributes in myotube-contacting neurites, is increased by NT3 [87]. Interestingly, similar to our findings in the p75^NTR^ null mice, NT3+/− mice exhibit impaired muscle contraction force and lower synaptic vesicle recycling in motor axon terminals [73]. We also found that p75^NTR^−/− mice displayed increased susceptibility to fatigue after tetanic nerve stimulation, a phenotype similar to that observed in NT4−/− mice [3]. The idea that Trk receptors and p75^NTR^ exert comparable effects at the NMJ is also supported by the functional consequences of reducing TrkB and p75^NTR^. Indeed, heterozygous TrkB+/− mice show decreased contractile force and muscle fiber CSA [44], as we found in p75^NTR^−/− mice. In turn, activation of TrkB signaling in mice null for TrkB.t1, an endogenous truncated dominant-negative variant of the receptor, results in increased isometric contraction force and increased CSA [17], exactly opposite to what we found in p75^NTR^−/− mice. An interesting comparison also emerges regarding TrkB and p75^NTR^ inhibition in a pathological context. Indeed, inhibition of TrkB expression in motor neurons of an amyotrophic lateral sclerosis mice model results in beneficial delaying effects on disease progression [97], whereas TrkB deletion impairs NMJ structure and function [28, 44]. Together with our findings, these results reveal the need for future research to elucidate how Trk and p75^NTR^ receptors act at the mature NMJ and how the signaling pathways controlled by these receptors are balanced to contribute to the correct apposition between the nerve terminal and the post-synaptic muscle domain. Our findings also contribute to a more comprehensive view of the effects that therapeutic attempts to target p75^NTR^ may have at the neuromuscular connectivity.

## Conclusion

We conclude that p75^NTR^ plays essential roles in neurotransmitter availability and on the organization of mature NMJs. Our results in p75^NTR^ exon III null mice describing uncharacterized phenotypes at the levels of synaptic function, muscle fiber structure, force production, and locomotor performance should be considered in the development of therapeutic strategies focused on targeting p75^NTR^ for NMJ repair.

## Additional files

Additional file 1: **Figure S1.** Gross NMJ organization of the LAL muscle from p75^NTR^−/− and control mice**.** Whole-mounts of LAL muscles of 2 months old WT and p75^NTR^−/− mice were stained to reveal presynaptic motor terminals (2H3 plus SV2 antibodies, red), postsynaptic AChRs (BTX, green) and terminal Schwann cells (S-100 antibody, white). The LAL muscle is innervated by a posterior auricular branch of the facial nerve. This profile generates five different rostral (R1-R5) and two caudal (C1-C2) innervation zones. The thin caudal muscle band has two clusters of NMJs (C1 and C2), located at medial and lateral ends of the muscle. The thicker rostral muscle band bears five clusters of NMJs (R1–R5), which are arranged in two groups (R1 and R2 together and R3-R5 together) [61]. Low magnification epifluorescence images of the right hemi-LAL were reconstructed and the rostral (R1-R5) and caudal (C1-C2) innervation regions were designated. Around 50 epifluorescence images were processed using the MosaicJ plugin of ImageJ. Bar: 500 μm. (PDF 1062 kb)

Additional file 2: **Figure S2.** p75^NTR^−/− mice muscles do not display molecular markers of denervation, degeneration/regeneration, or atrophy. TA muscle cryosections from WT and p75^NTR^−/− mice were labeled with antibodies (green) to detect myogenin- (a, arrows) or eMyHC-positive fibers (b, arrows). Nuclei were counterstained with DAPI (a). Positive control cryosections were obtained from denervated (a) or barium chloride-treated (b) TA muscles from control mice. Bar: 50 μm. (c) Total protein samples of TA muscles from WT and p75^NTR^−/− mice were analyzed by Western blot using specific antibodies to detect MuRF-1 and Atrogin-1. Control TA muscle protein samples were obtained from adult WT mice treated with angiotensin II, as described [60]. The levels of β-actin were used as loading control. (PDF 8535 kb)

Additional file 3: **Figure S3.** Three-dimensional projection of NMJs from p75^NTR^−/− and control mice muscles. Diaphragm muscles from 2-months-old WT and p75^NTR^−/− mice were stained with BTX to reveal AChR aggregates. Representative 3D images of NMJs from WT and p75^NTR^−/− mice. Images were obtained by processing confocal z-stack images using the Imaris software. The color map indicates the volume of NMJs, from 464 (blue) to 6374 μm^3^ (red). Bar: 50 μm. (PDF 1259 kb)

Additional file 4: **Figure S4.** Unaltered levels of BDNF and TrkB in the sciatic nerve and the spinal cord of p75^NTR^−/− mice**.** (a) Sciatic nerve cryosections from WT and p75^NTR^−/− mice were labeled with antibodies to detect BDNF. Bar: 10 μm. Similar levels of BDNF were detected mainly in the cell body of Schwann cells (arrowheads). Total protein samples of the sciatic nerve (b) or the spinal cord (c) from WT and p75^NTR^−/− mice were analyzed by Western blot using specific antibodies to detect TrkB or BDNF. The levels of β-actin and GAPDH were used as loading controls. (PDF 784 kb)

## Data Availability

The datasets used and/or analyzed during the current study available from the corresponding author on reasonable request.

## Abbreviations

ACh: Acetylcholine
AChE: Acetylcholinesterase
AChRs: Acetylcholine receptors
BDNF: Brain-derived neurotrophic factor
BTX: α-Bungarotoxin
CMAP: Compound muscle action potential
CSA: Cross-sectional area
KI: Kyphotic index
LAL: *Levator auris longus*
MyHC: Myosin heavy chain
NADH-TR: NADH-thioreductase
NGF: Nerve growth factor
NMJ: Neuromuscular junction
NT: Neurotrophin
P75^NTR^: P75 neurotrophin receptor
RNS: Repetitive nerve stimulation
RRP: Readily releasable pool
TA: *Tibialis anterior*
TnC: Troponin C
Trks: Tyrosine kinase receptors
