# Supplementary figures and images for: The p75NTR neurotrophin receptor is required to organize the mature neuromuscular synapse by regulating synaptic vesicle availability
Source: Acta Neuropathol Commun. 2019 Sep 12;7:147. doi: 10.1186/s40478-019-0802-7 (PMC6739937; doi:10.1186/s40478-019-0802-7)

Additional file 1

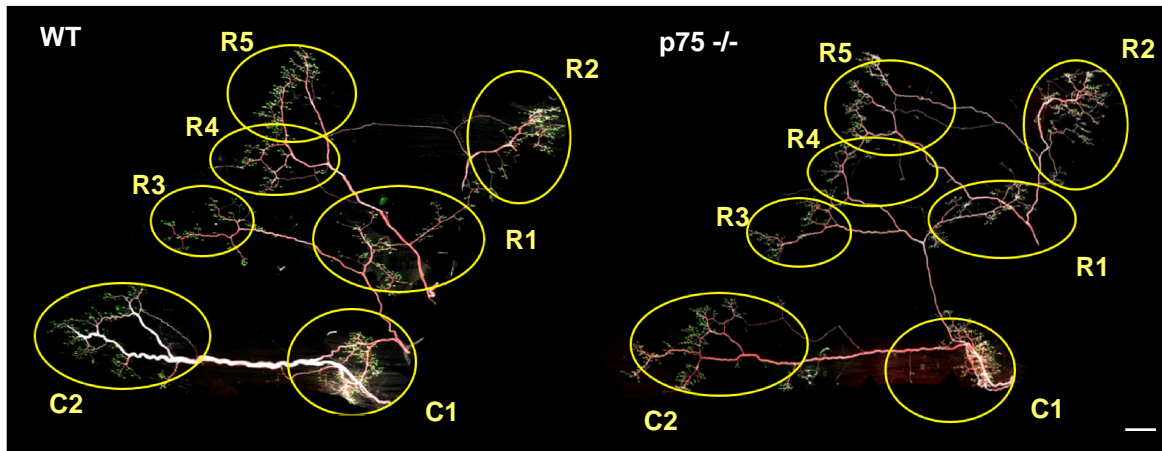

Supplement: Supplementary file 1 — Figure S1. Gross NMJ organization of the LAL muscle from p75NTR−/− and control mice. Whole-mounts of LAL muscles of 2 months old WT and p75NTR−/− mice were stained to reveal presynaptic motor terminals (2H3 plus SV2 antibodies, red), postsynaptic AChRs (BTX, green) and terminal Schwann cells (S-100 antibody, white). The LAL muscle is innervated by a posterior auricular branch of the facial nerve. This profile generates five different rostral (R1-R5) and two caudal (C1-C2) innervation zones. The thin caudal muscle band has two clusters of NMJs (C1 and C2), located at medial and lateral ends of the muscle. The thicker rostral muscle band bears five clusters of NMJs (R1–R5), which are arranged in two groups (R1 and R2 together and R3-R5 together) [61]. Low magnification epifluorescence images of the right hemi-LAL were reconstructed and the rostral (R1-R5) and caudal (C1-C2) innervation regions were designated. Around 50 epifluorescence images were processed using the MosaicJ plugin of ImageJ. Bar: 500 μm. (PDF 1062 kb) [file 40478_2019_802_MOESM1_ESM.pdf]

## Additional file 2

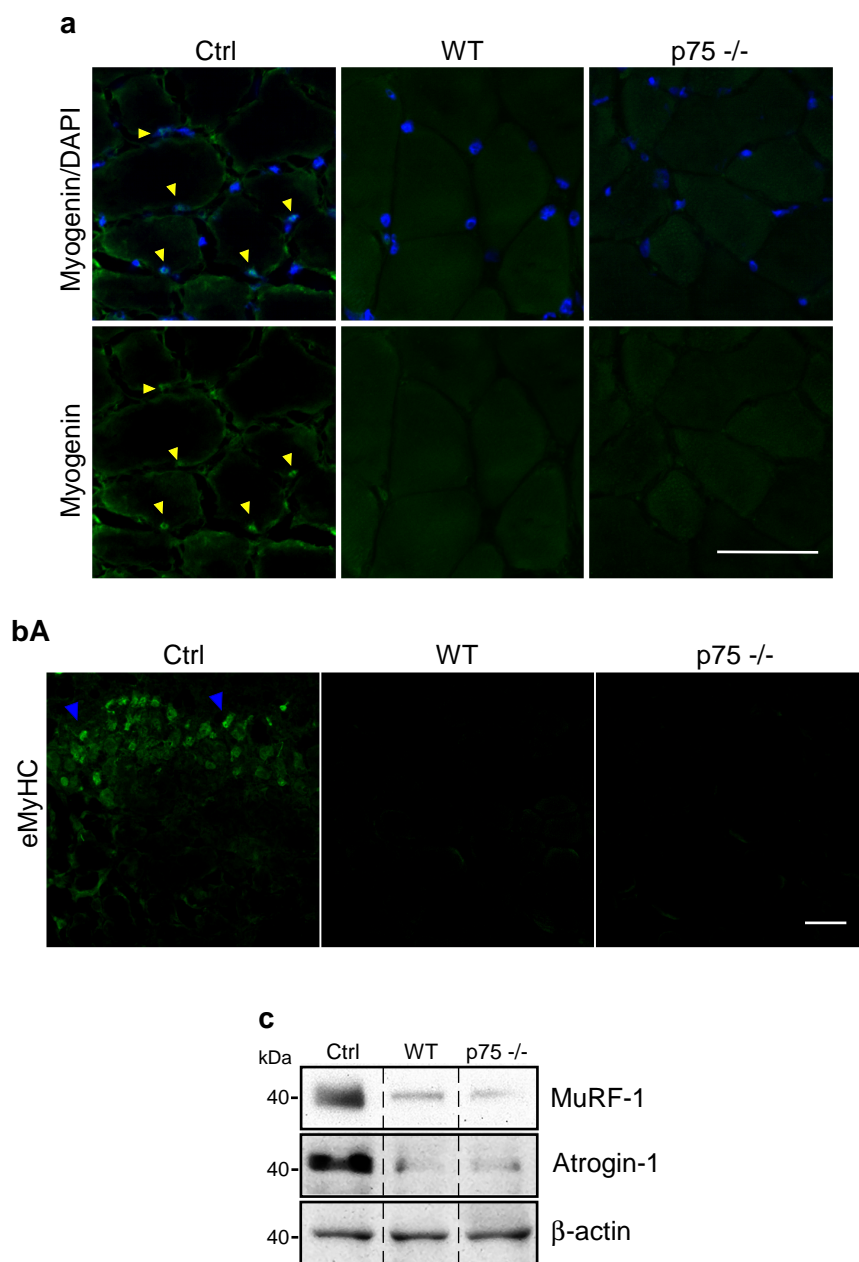

Supplement: Supplementary file 2 — Figure S2. p75NTR−/− mice muscles do not display molecular markers of denervation, degeneration/regeneration, or atrophy. TA muscle cryosections from WT and p75NTR−/− mice were labeled with antibodies (green) to detect myogenin- (a, arrows) or eMyHC-positive fibers (b, arrows). Nuclei were counterstained with DAPI (a). Positive control cryosections were obtained from denervated (a) or barium chloride-treated (b) TA muscles from control mice. Bar: 50 μm. (c) Total protein samples of TA muscles from WT and p75NTR−/− mice were analyzed by Western blot using specific antibodies to detect MuRF-1 and Atrogin-1. Control TA muscle protein samples were obtained from adult WT mice treated with angiotensin II, as described [60]. The levels of β-actin were used as loading control. (PDF 8535 kb) [file 40478_2019_802_MOESM2_ESM.pdf]

Additional file 3

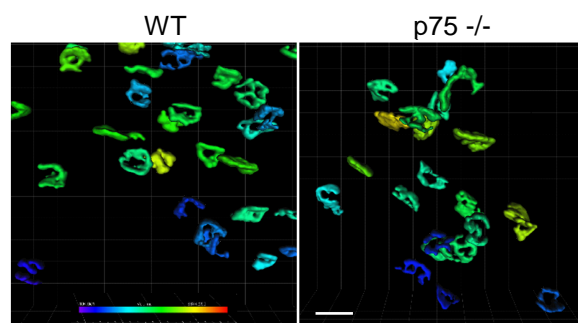

Supplement: Supplementary file 3 — Figure S3. Three-dimensional projection of NMJs from p75NTR−/− and control mice muscles. Diaphragm muscles from 2-months-old WT and p75NTR−/− mice were stained with BTX to reveal AChR aggregates. Representative 3D images of NMJs from WT and p75NTR−/− mice. Images were obtained by processing confocal z-stack images using the Imaris software. The color map indicates the volume of NMJs, from 464 (blue) to 6374 μm3 (red). Bar: 50 μm. (PDF 1259 kb) [file 40478_2019_802_MOESM3_ESM.pdf]

## Additional file 4

**a**

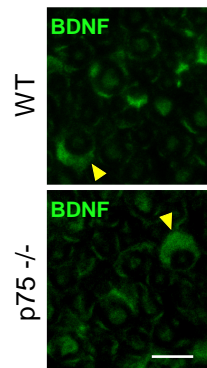

**b**

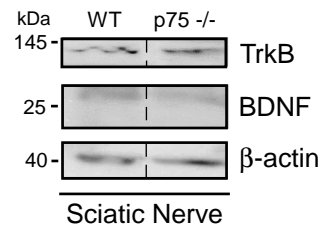

**c**

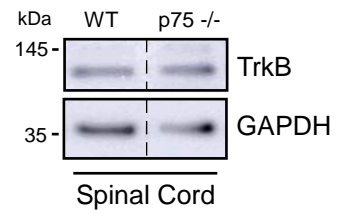

Supplement: Supplementary file 4 — Figure S4. Unaltered levels of BDNF and TrkB in the sciatic nerve and the spinal cord of p75NTR−/− mice. (a) Sciatic nerve cryosections from WT and p75NTR−/− mice were labeled with antibodies to detect BDNF. Bar: 10 μm. Similar levels of BDNF were detected mainly in the cell body of Schwann cells (arrowheads). Total protein samples of the sciatic nerve (b) or the spinal cord (c) from WT and p75NTR−/− mice were analyzed by Western blot using specific antibodies to detect TrkB or BDNF. The levels of β-actin and GAPDH were used as loading controls. (PDF 784 kb) [file 40478_2019_802_MOESM4_ESM.pdf]
